# Antigen Specificity and Clinical Significance of IgG and IgA Autoantibodies Produced *in situ* by Tumor-Infiltrating B Cells in Breast Cancer

**DOI:** 10.3389/fimmu.2018.02660

**Published:** 2018-11-20

**Authors:** Soizic Garaud, Pawel Zayakin, Laurence Buisseret, Undine Rulle, Karina Silina, Alexandre de Wind, Gert Van den Eyden, Denis Larsimont, Karen Willard-Gallo, Aija Linē

**Affiliations:** ^1^Molecular Immunology Unit, Institut Jules Bordet, Universite Libre de Bruxelles, Brussels, Belgium; ^2^Cancer Biomarker and Immunotherapy Group, Latvian Biomedical Research and Study Centre, Riga, Latvia; ^3^Institute of Pathology and Molecular Pathology, University Hospital Zurich, Zurich, Switzerland; ^4^Institute of Experimental Immunology, University of Zurich, Zurich, Switzerland; ^5^Department of Pathology, Institut Jules Bordet, Université Libre de Bruxelles, Brussels, Belgium; ^6^Translational Cancer Research Unit Antwerp, Oncology Centre, General Hospital Sint Augustinus, Wilrijk, Belgium; ^7^Faculty of Biology, University of Latvia, Riga, Latvia

**Keywords:** tumor-infiltrating B cells, autoantibodies, IgG, IgA, tertiary lymphoid structures, breast cancer

## Abstract

An important role for tumor infiltrating B lymphocytes (TIL-B) in the immune response to cancer is emerging; however, very little is known about the antigen specificity of antibodies produced *in situ*. The presence of IgA antibodies in the tumor microenvironment has been noted although their biological functions and clinical significance are unknown. This study used a 91-antigen microarray to examine the IgG and IgA autoantibody repertoires in breast cancer (BC). Tumor and adjacent breast tissue supernatants and plasma from BC patients together with normal breast tissue supernatants and plasma from healthy controls (patients undergoing mammary reduction and healthy blood donors) were analyzed to investigate relationships between autoantibodies and the clinical, histological and immunological features of tumors. Our data show that >84% of the BC samples tested contain autoantibodies to one or more antigens on the array, with ANKRD30BL, COPS4, and CTAG1B being most frequently reactive. *Ex vivo* TIL-B responses were uncoupled from systemic humoral responses in the majority of cases. A comparison of autoantibody frequencies in supernatants and plasma from patients and controls identified eight antigens that elicit BC-associated autoantibody responses. The overall prevalence of IgG and IgA autoantibodies was similar and while IgG and IgA responses were not linked they did correlate with distinct clinical, pathological and immunological features. Higher levels of *ex vivo* IgG responses to BC-associated antigens were associated with shorter recurrence-free survival (RFS), HER2 overexpression and lower tumor-infiltrating CD8^+^ T cell counts. Higher IgA levels were associated with estrogen and progesterone receptor-negative cancers but were not significantly associated with RFS. Furthermore, *ex vivo* IgA but not IgG autoantibodies reactive to BC-associated antigens were linked with germinal center and early memory B cell maturation and the presence of tertiary lymphoid structures suggesting that these TIL-B are activated in the tumor microenvironment. Overall, our results extend the current understanding of the antigen specificity, the biological and the clinical significance of IgG and IgA autoantibodies produced by BC TIL-B *in situ*.

## Introduction

Breast cancer (BC) is a profoundly heterogeneous disease not only in respect of its molecular architecture and clinical course but also to the hosts' immune responses against the tumor (1). The intensity and mode of anti-tumor immune response depends on the cellular composition, density, functional phenotype and spatial distribution of tumor-infiltrating immune cells (2). While higher density of tumor-infiltrating CD8^+^ T cells is consistently associated with favorable clinical outcomes in various solid tumors, including BC (3, 4), the functional role and clinical significance of tumor-infiltrating B lymphocytes (TIL-B) is far less clear (5). Several studies have shown that higher number of CD20^+^ TIL-B is associated with better BC specific survival and longer recurrence-free survival (RFS) (6–8), or predicts pathological complete response in BC patients treated with neoadjuvant chemotherapy (9, 10). On the contrary, higher counts of CD138^+^ B lymphocytes and plasma cells (PC) were associated with poor cancer-specific survival in invasive ductal BC (6, 11), whereas higher counts of CD38^+^ PC have been found to be associated with better disease-free survival in triple negative BC (12). Another recent study showed that higher CD19^+^ and CD20^+^ TIL-B but not CD138^+^ PC counts are associated with larger tumor size and shorter RFS in patients with ductal carcinoma *in situ* (13). At the same time, a meta-analysis of gene expression signatures in ~18,000 various human tumors revealed PC-gene expression signature as one of the most significant predictors of favorable survival across various solid tumors (14). The reasons for these controversies are poorly understood so far.

Important aspect for gaining a better understanding into the TIL-B effector functions is the antigen specificity of the antibodies they produce. Most of the studies addressing the repertoire of autoantibodies have been focused on plasma or serum IgG class antibodies, while the repertoire of antibodies produced *in situ* by TIL-B is poorly characterized. Moreover, although the presence of high amounts of IgA class antibodies in BC tissues has been reported (15), the antigen specificity and functions of IgAs has not been studied so far. Earlier studies of TIL-B antigen specificity were based on the analysis of immunoglobulin genes and suggested that TIL-B undergo antigen-driven oligoclonal proliferation, somatic hypermutation and affinity maturation *in situ* (16–18). However, only a few antigens recognized by TIL-B have been identified in BC so far and include the cytoskeletal protein β-actin (19), ganglioside D3 (20), and CEA, MUC1, and FN1 (ED-B domain) (21).

In the current study, we characterized the repertoire of IgG and IgA autoantibodies present in the BC and normal breast tissue supernatants and plasma of BC patients and controls using our in-house 91-antigen microarrays, and analyzed the relationship between the presence of autoantibodies and clinical, histological and immunological features of tumors.

## Materials and methods

### Samples and study population

Paired tumor and adjacent breast tissue specimens and plasma samples were collected from 66 primary BC patients diagnosed and treated at the Institut Jules Bordet, Brussels between July 2012 and October 2013 and followed up until January 2018. Inclusion criteria: untreated invasive primary carcinomas, stage I to III at diagnosis. The characteristics of the study population are provided in Table 1. Normal breast tissue specimens were collected from 23 women undergoing mammary reduction surgery. Fresh breast tissues were obtained at the day of surgery and dissociated (without enzymatic digestion) using the GentleMacs Dissociator (Miltenyi Biotec) (22). The resulting cell suspension was filtered following each dissociation run using a 40 μm cell strainer (BD Falcon), washed with X-VIVO 20, centrifuged 15 min at 600 g, and resuspended in X-VIVO 20 before flow cytometric analysis. The tissue supernatant was the initial 3 ml of X-VIVO 20 recovered after the first round of dissociation, which was subsequently clarified by centrifugation for 15 min at 13,000 g.

**Table 1:** Association of the IgG-TAA and IgA-TAA scores with clinicopathological parameters.

**Parameter**	**Mean IgG-TAA score**	**Mean IgA-TAA score**	**Parameter**	**Mean IgG-TAA score**	**Mean IgA-TAA score**
**Age**			**PR status**		
<50 years, *n* = 17	0.68	0.44	PR+, *n* = 45	0.5	0.36
≥50 years, *n* = 49	0.90	0.52	PR–, *n* = 21	1.57	0.81
M-WU, *P*-value	0.66	0.96	M-WU, *P*-value	0.22	**0.026**
**Histology**			**HER2 status**		
Ductal, *n* = 51	0.87	0.53	HER2+, *n* = 12	1.34	0.66
Lobular, *n* = 13	0.83	0.43	HER2–, *n* = 53	0.74	0.47
M-WU, *P*-value	0.69	0.27	M-WU, *P*-value	**0.032**	0.23
**Subtype**			**Ki67 status**		
Lobular, *n* = 11	0.71	0.35	<20, *n* = 37	0.55	0.40
Luminal A, *n* = 23	0.49	0.43	≥20, *n* = 29	1.21	0.63
Luminal B, *n* = 15	0.28	0.31	M-WU, *P*-value	0.50	0.14
HER2+, *n* = 12	1.34	0.66	**Tumor size, mm**		
TN, *n* = 5	3.28	1.36	<20, *n* = 37	0.51	0.48
KW, *P*-value	0.13	0.22	20–49, *n* = 26	1.32	0.58
**Stage**			≥50, *n* = 3	0.78	0.12
I, *n* = 29	0.29	0.40	KW, *P*-value	0.30	0.58
II, *n* = 29	1.29	0.60	**Positive lymph nodes**		
III, *n* = 7	1.1	0.31	0, *n* = 39	0.86	0.55
KW, *P*-value	0.29	0.71	1–4, *n* = 20	0.71	0.47
**Grade**			≥5, *n* = 6	1.28	0.36
1, *n* = 10	0.47	0.42	KW, *P*-value	0.23	0.91
2, *n* = 24	0.50	0.31	***in situ***		
3, *n* = 31	1.25	0.69	Yes, *n* = 48	0.75	0.49
KW, *P*-value	0.26	**0.044**	No, *n* = 15	0.96	0.48
**ER status**			M-WU, *P*-value	0.99	0.69
ER+, *n* = 57	0.60	0.4			
ER–, *n* = 9	2.4	1.12			
M-WU, *P*-value	0.25	**0.027**			

*M-WU, Mann-Whitney U test, KW, Kruskal-Wallis test. Statistically significant findings are highlighted in bold*.

Plasma samples from 200 cancer-free women were obtained from the Latvian Genome Database (Riga cohort) and additional 18 plasma samples were collected from cancer-free women at the Institute Jules Bordet (Brussels cohort). Plasma samples from the BC patients were collected before the surgery. The blood samples were collected in EDTA-treated tubes (Riga cohort) or heparin-treated tubes (Brussels cohort) and processed within 24 h. The blood tubes were centrifuged at 200 *g* for 10 min, the plasma was aliquoted and stored at −80°C until use.

The study was conducted according to the Declaration of Helsinki. The specimens were collected after the patients' informed written consent was obtained and the research on anonymized patients' samples was approved by the Institut Jules Bordet's Medical Ethics Committee (Accepted project CE 1981) and the ethical committee of the University of Latvia, Institute of Experimental and Clinical Medicine.

### Immunohistochemistry (IHC)

Formalin-fixed paraffin-embedded tissue sections (4 μm) were immunohistochemically stained for CD3 (pan T cells; Dako) and CD20 (pan B cells; Dako) on a Ventana Benchmark XT automated staining instrument (Ventana Medical Systems). A detailed protocol for the dual CD3/CD20 immunohistochemical stain is described in Buisseret et al. (23).

### Flow cytometry

Cell suspensions were incubated with manufacturer's suggested dilutions of fluorescently labeled primary monoclonal antibodies (Supplementary Table S1) for 1 h at 4°C in 100 μl of X-VIVO 20 followed by washing with PBS. After washing once, a lysing solution was used for the lysis of red blood cells in cell suspension from breast tissues (VersaLyse, BC). Fluorescently labeled cells were acquired on a GALLIOS 10/3 cytometer and the data were analyzed using Kaluza Flow Cytometry Analysis v1.2 software.

### Production and processing of antigen microarray

For the production of antigen array, cDNA fragments encoding 91 antigens were cloned into the pFN19A (HaloTag®7) T7 SP6 Flexi bacterial expression vector (Promega, USA) as described before (24). A His-tag coding sequence was introduced upstream the Halo-tag and the cDNAs encoding the antigens were inserted at the 3′ end of the Halo tag. The recombinant proteins were expressed in *E. coli* XL Blue cells, purified using Ni-TED columns (Macherey-Nagel, Germany) and quality-checked by Western blot analysis. The proteins were diluted to 0.03–0.1 mg/ml in PBS, 2% glycerol and printed on nickel chelate coated glass slides (Xenopore) in duplicates using QArray Mini microarray printer (Genetix, UK). His-Halo tags were used as negative controls.

The slides were processed as described by Zayakin et al. with some modifications (25). Briefly, the slides were blocked in 7% (w/v) milk powder in TBS, 0.05% Tween-20 and incubated with 1:2 diluted tissue culture supernatants or 1:50 diluted plasma samples that had been pre-absorbed with His-Halo-tag expressing *E. coli* lysates. The slides were washed overnight and incubated with 1:500 diluted rabbit anti-Halo-tag antibody (G9281, Promega, USA). IgG and IgA autoantibodies were detected with Cy5-conjugated mouse anti-human IgG secondary antibody (1:2,000) (109-175-098, Jackson ImmunoResearch, USA) and Cy5-conjugated goat anti-human IgA secondary antibody (1:750) (109-605-011, Jackson ImmunoResearch, USA), respectively, while the anti-Halo tag antibodies were detected with Cy3-conjugated goat anti-rabbit IgG secondary antibody (1:2,000) (111-165-046, Jackson ImmunoResearch, USA).

The arrays were scanned at 10 μm resolution in PowerScanner (Tecan, Switzerland). The results were recorded as TIFF files and the raw data were processed with GenePix™ Pro 4.0 software (Molecular Devices, USA) and further analyzed using an *ad hoc* program composed in R language.

### Microarray data processing and statistical analyses

For each antigen, the mean Cy5 and Cy3 signals were background subtracted and the Cy5/Cy3 ratios were averaged between replicates. Next, the Cy5/Cy3 values were normalized by applying quantile normalization to 25th and 75th percentiles between all samples. In order to define reactive antigens in each array, the values were log transformed and the cut-off value was set at median plus 10 SDs of trimmed (25th to 75th percentiles) signals in each sample. This cut-off could clearly and reproducibly distinguish background reactivity from the reactive antigens (outliers deviating from normal distribution). The frequencies of reactive antigens were calculated in each group of samples. BC-associated antigens were defined by comparing combined IgG and IgA reactivity in SN-BC and PB-BC samples vs. SN-MR and PB-HD samples. The Fisher's exact test and was applied to determine the level of significance and Benjamini-Hochberg (BH) procedure was used for adjusting the false discovery rate for multiple comparisons. In order to assess the combined reactivity against BC-associated antigens, IgG-TAA and IgA-TAA scores were calculated by summing of signal intensities (above the cut-off) of all BC-associated antigens in each sample.

Statistical analyses were performed using an *ad hoc* program composed in R language. The prognostic significance of IgG-TAA and IgA-TAA scores and B cell counts was assessed by the Kaplan-Meier survival curve and the Log-rank test. The relapse-free survival was calculated from the date of surgery till diagnosis of relapse. Patients who were alive on the last day of follow-up or died of BC non-related cause were censored.

The Mann-Whitney *U* and Kruskal-Wallis tests were used to compare the IgG and IgA reactivity between the groups of patients stratified by clinical or histological parameters. Student's *t*-test was used to compare quantitative clinical, histological and immunological variables. The Fisher's exact test was used for categorical variables between the groups of patients dichotomized based on the presence/absence of the given autoantibodies or low and high TAA scores (divided by the median). The two-tailed Spearman rank test was used to analyze the correlation between continuous variables.

## Results

### Detection of IgG and IgA class autoantibodies in tissue supernatants and plasma

In order to examine the repertoire of autoantibodies produced by the TIL-B, the 91-antigen arrays were tested for the reactivity with IgG and IgA autoantibodies in the tumor and adjacent normal breast tissue supernatants and plasma samples from BC patients and healthy controls. Workflow of the study is shown in Figure S1. The microarray comprised fragments of 91 antigens, including cancer-testis antigens, such as CTAG1B, MAGEA1, MAGEA3, DDX53, CTAGE5, SPAG8, SPAG6, and CSAG3, other known tumor antigens, such as TP53, MUC1, SOX2, BRCA2, TERT and BIRC5, and artificial peptides previously shown to elicit cancer-associated autoantibody responses (25–27). Complete list of antigens is provided in Supplementary Table S2. IgG were tested in the tumor and adjacent normal breast tissue supernatants (denoted by SN-BC and SN-AT, respectively) and plasma samples from 66 BC patients (denoted by PB-BC), normal breast tissue supernatant samples from 23 healthy women undergoing mammary reduction surgery (denoted by SN-MR) and plasma samples from 200 (Riga cohort) and 18 (Brussels cohort) healthy women (denoted by PB-HD). IgA were tested in the same sample sets, except for PB-HD Riga cohort.

To assess which of the autoantibodies are BC-associated, the frequencies of combined IgG and IgA responses in SN-BC and PB-BC samples were compared against those in SN-MR and PB-HD sample sets and eight antigens with BH adj. *P*-values <0.05 were considered to be significantly associated with BC (Table 2). Among them were several previously known cancer antigens as well as novel peptides. CTAG1B, BRCA2, and SPAG8 have been previously shown to induce humoral immune response in BC (27–29) and other cancer patients (25, 27, 30), ANKRD30BL and COPS4 were previously found to elicit autoantibody response in gastric and thyroid cancer and melanoma (24, 26). Artificial peptides AP1292, AP1775 and AP1361 were reacting with sera from gastric cancer patients in our previous studies (24) and may represent neoantigens or mimotopes of other cancer-associated antigens.

**Table 2:** Frequencies of IgG and IgA responses in tumor and normal breast tissue supernatants and plasma samples, %.

**Antigen**	**SN-BC**		**SN-AT**		**PB-BC**		**SN-MR**		**PB-HD**		**SN-BC +PB-BC^#^**	**SN-MR +PB-HD^#^**	***P*-value**	**BH adj. *P*-value**
	**IgG**	**IgA**	**IgG**	**IgA**	**IgG**	**IgA**	**IgG**	**IgA**	IgG^*^	IgA^**^				
**ANKRD30BL**	**20.93**	**48.78**	**16.67**	**36.59**	**12.5**	**10**	**17.65**	**20**	**2.34**	**14.29**	**22.67**	**5.28**	**1.20E-07**	**5.46E-06**
**COPS4**	**27.27**	**18.18**	**20.31**	**6.25**	**7.58**	**3.03**	**22.73**	**4.35**	**0.46**	**0**	**14.02**	**2.5**	**4.70E-07**	**1.43E-05**
**CTAG1B**	**30.77**	**7.69**	**25**	**9.38**	**21.21**	**34.85**	**8.7**	**4.35**	**1.87**	**5.56**	**23.66**	**2.88**	**9.00E-14**	**8.19E-12**
AP1735	16.67	21.21	23.81	39.06	66.67	72.73	26.09	34.78	32.09	58.82	44.32	33.45	0.011	0.091
AP1588	21.21	13.64	23.44	18.75	45.45	62.12	21.74	34.78	24.23	72.22	35.61	28.29	0.076	0.288
**SPAG8**	**13.64**	**15.38**	**20.31**	**15.62**	**40**	**37.88**	**8.7**	**13.04**	**13.48**	**16.67**	**26.72**	**13.22**	**1.60E-04**	**0.002**
AP1743	17.46	9.23	17.46	9.52	32.31	19.7	4.35	0	31.49	11.11	19.69	24.49	0.2	0.606
**AP1292**	**20.31**	**3.08**	**11.67**	**1.59**	**1.56**	**0**	**0**	**0**	**0**	**0**	**6.2**	**0**	**6.30E-06**	**1.43E-04**
AP799	14.06	9.09	15.87	11.11	31.82	27.27	26.09	21.74	14.29	27.78	20.61	16.73	0.27	0.664
**AP1775**	**16.67**	**6.06**	**15.62**	**6.25**	**55.38**	**42.42**	**13.04**	**4.35**	**14.78**	**16.67**	**30.04**	**13.86**	**8.60E-06**	**1.57E-04**
AP463	10.61	4.55	12.5	9.38	25.76	24.24	26.09	21.74	8.87	22.22	16.29	12.36	0.22	0.625
AP798	9.09	4.55	10.94	6.25	21.21	15.15	8.7	0	6.54	22.22	12.5	7.19	0.043	0.225
AP1654	7.58	4.55	9.38	6.25	20	13.64	13.04	8.7	10.19	22.22	11.41	11.07	1	1
CSAG3	6.45	4.92	1.61	5	9.68	9.84	10	0	3.23	5.88	7.72	3.64	0.055	0.236
**AP1361**	**1.52**	**9.23**	**1.56**	**9.38**	**3.03**	**6.06**	**0**	**4.35**	**0**	**0**	**4.94**	**0.36**	**6.40E-04**	**0.007**
DNAAF1	4.55	6.06	6.25	9.38	44.62	45.45	21.74	52.17	11.57	44.44	25.1	17.86	0.046	0.225
SPAG6	0	10.2	5.13	7.69	4.08	6.67	0	5.56	0	6.25	5.56	1.31	0.046	0.225
MYC	4.55	4.55	1.56	3.12	18.18	21.21	8.7	0	4.25	44.44	12.12	6.88	0.04	0.225
AP383	6.06	3.03	7.81	3.12	16.67	15.15	4.35	0	8.41	11.11	10.23	7.55	0.29	0.677
TYR	6.06	3.03	9.38	3.12	15.38	12.12	4.35	0	10.19	11.11	9.13	8.89	1	1
**BRCA2**	**3.08**	**4.62**	**3.17**	**3.12**	**16.67**	**27.42**	**4.35**	**17.39**	**1.39**	**6.67**	**12.79**	**3.25**	**3.90E-05**	**5.92E-04**
ASB9	4.55	3.03	6.25	3.12	13.85	10.61	0	4.35	5.35	5.56	6.82	3.56	0.15	0.47
SOX2	4.62	1.52	3.23	3.17	19.7	10.61	0	0	4.55	5.56	9.13	3.82	0.02	0.14
AP1795	4.55	1.52	4.69	3.12	3.03	1.52	4.35	8.7	2.31	5.56	2.65	3.38	0.79	1
AP1650	6.06	0	1.56	1.56	4.55	4.55	0	4.35	2.36	11.11	3.79	2.9	0.64	0.987

* Tested with Riga and Brussels PB-HD cohorts;

** Tested with Brussels PB-HD cohort only,

# *Combined IgG and IgA frequencies. AP, artificial peptide. Antigens showing BC-associated reactivity pattern are marked in bold. Antigens with significantly different (Fisher test P-value <0.01) IgG vs. IgA reactivity in SN-BC are underlined*.

In total, 86.4% of SN-BC samples had IgG and ~84.8%–IgA autoantibodies against one or more antigens included in the antigen array. The most frequently reacting antigens (ranked by the frequency of combined IgG and IgA responses in SN-BC samples) are listed in Table 2 and include both BC-associated antigens and BC non-related antigens. The frequencies of autoantibodies in SN-AT samples were only slightly lower than in SN-BC samples, however it is not clear if these antibodies are produced *ex-vivo* by B cells present in the adjacent normal tissues or have diffused from the tumor tissues. Most of these antibodies, except for anti-AP1292 and SOX2, were also found in SN-MR samples, though at lower frequencies.

### Prevalence of IgG and IgA class autoantibodies in tissues and plasma of BC patients

**Figure 1:**
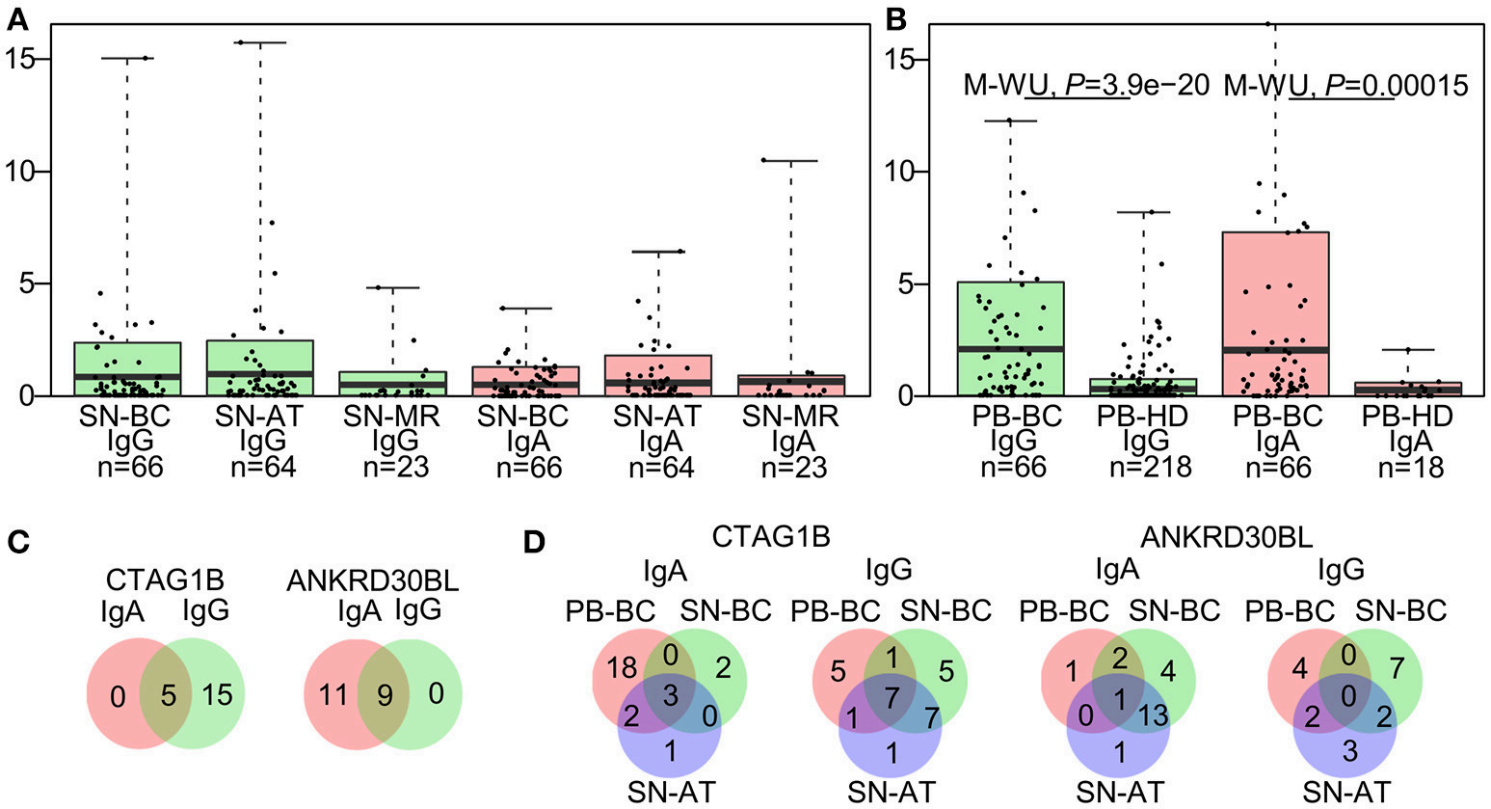
Characteristics of the autoantibody prevalence in breast tissue supernatants and plasma. **(A)** IgG-TAA and IgA-TAA scores in tissue supernatants and **(B)** plasma samples. Box plots show mean and 0.1–0.9 quantiles, whiskers show maximal values, dots represent individual samples. The Mann-Whitney *U* test was used to compare the scores between PB-BC and PB-HD, and Kruskal-Wallis test was used to compare the scores among SN-BC, SN-AT and SN-MR samples. **(C)** Venn diagrams showing number of patients with concordant or mutually exclusive IgG and IgA responses to CTAG1B and ANKRD30BL in SN-BC samples. **(D)** Venn diagrams showing the prevalence of anti-CTAG1B and anti-ANKRD30BL IgG and IgA antibodies in SN-BC, SN-AT, and PB-BC samples from BC patients.

To assess combined levels of BC-associated autoantibody production, IgG-TAA and IgA-TAA scores were calculated for each of the SN-BC samples based on the signal intensities against the BC-associated antigens. The mean scores in tissue supernatant and plasma samples are shown in Figures 1A,B, respectively.

As shown in Table 2, the majority of antigens were recognized by both IgG and IgA autoantibodies at similar frequencies, except for 3 antigens. CTAG1B and AP1292 more frequently elicited IgG responses, while ANKRD30BL–IgA responses (Fisher's exact test *P*-value <0.01). Furthermore, a fraction of patients mounted both IgG and IgA responses against the same antigen, while others produced exclusively IgG or IgA class antibodies (selected antigens shown in Figure 1C; all BC-associated antigens shown in Figure S2).

To assess whether the occurrence of autoantibodies in tissue supernatants is correlated with that in plasma, we compared the prevalence of IgG and IgA responses to each of the BC-associated antigens in SN-BC, SN-AT, and PB-BC samples. We found that only a minor fraction of patients had autoantibodies to a given antigen both in plasma and tissue supernatants, while in the majority of patients the presence of autoantibodies in the tissue supernatants was uncoupled from that in plasma (selected antigens shown in Figure 1D; all BC-associated antigens shown in Figure S3A and total frequencies of BC-associated IgG and IgA responses are shown in Figure S3B).

### Association of TIL-B antibodies with histological and clinical features

In order to gain an insight into the clinical significance of TIL-B-produced antibodies, the IgG-TAA and IgA-TAA scores were correlated with the clinical and histological features including histological type, subtype, stage and grade of BC, and hormone receptor status (Table 1).

**Figure 2:**
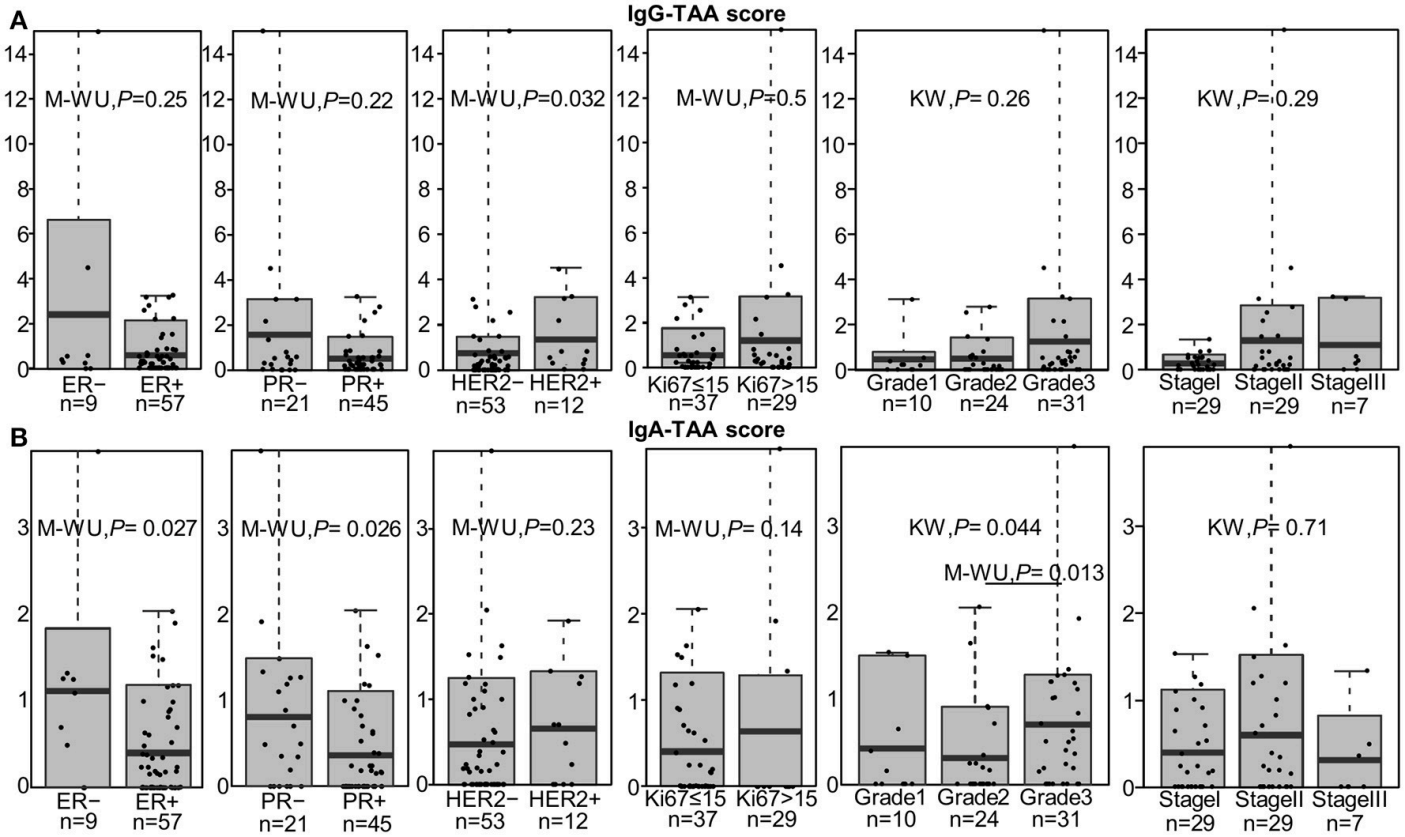
Association of the IgG-TAA and IgA-TAA scores with clinicopathological features. **(A)** IgG-TAA scores and **(B)** IgA-TAA scores in SN-BC samples from patients stratified according to hormone receptor status, Ki67 level, grade and stage. Box plots show mean and 0.1–0.9 quantiles, whiskers show maximal values, dots represent individual samples. M-WU, Mann-Whitney *U* test, KW, Kruskal-Wallis test.

IgG-TAA score was significantly higher in HER2-positive tumors as compared to HER2- negative cancers [mean fold change (FC) = 1.81; *P* = 0.032] and showed a tendency to be increased in estrogen receptor (ER) and progesterone receptor (PR) negative cancers (Figure 2A). Furthermore, the IgG-TAA score showed a tendency to increase with the grade and stage of the disease. IgA-TAA score was significantly higher in ER and PR-negative cancers than in the hormone receptor-positive cancers (mean FC = 2.80, *P* = 0.027, FC = 2.25, *P* = 0.028) and was higher in grade 3 than grade 2 cancers (mean FC = 2.22, *P* = 0.013; Figure 2B). The scores were not significantly associated with age, tumor size, number of positive lymph nodes, Ki67 levels nor differed between ductal and lobular BC.

### IgA production is linked to B cell maturation stages

To gain a better understanding into the phenotype of TIL-B producing autoantibodies *in situ*, we correlated the levels of IgG and IgA against individual antigens and the IgG and IgA-TAA scores with the density (cells/mm^3^ of tissue) of CD19^+^ B cells, plasmablasts/plasma cells (PB/PC), CD4^+^ and CD8^+^ T cells, and the maturation stage of B cells determined by flow cytometry. PB/PC were defined as CD45^+^CD19^+^CD27^+^CD38^++^ cells, naïve B cells were defined as CD19^+^CD27^−^ cells and memory B cells—as CD19^+^CD27^+^ cells. The gating strategies used to define these cell types and B cell maturation stages are shown in Figure S4. Additionally, the density of tertiary lymphoid structures (TLS) was determined by IHC and defined as the number of dense B-cell aggregates with an adjacent T-cell zone relative to the tumor area (per mm^2^) (23).

**Figure 3:**
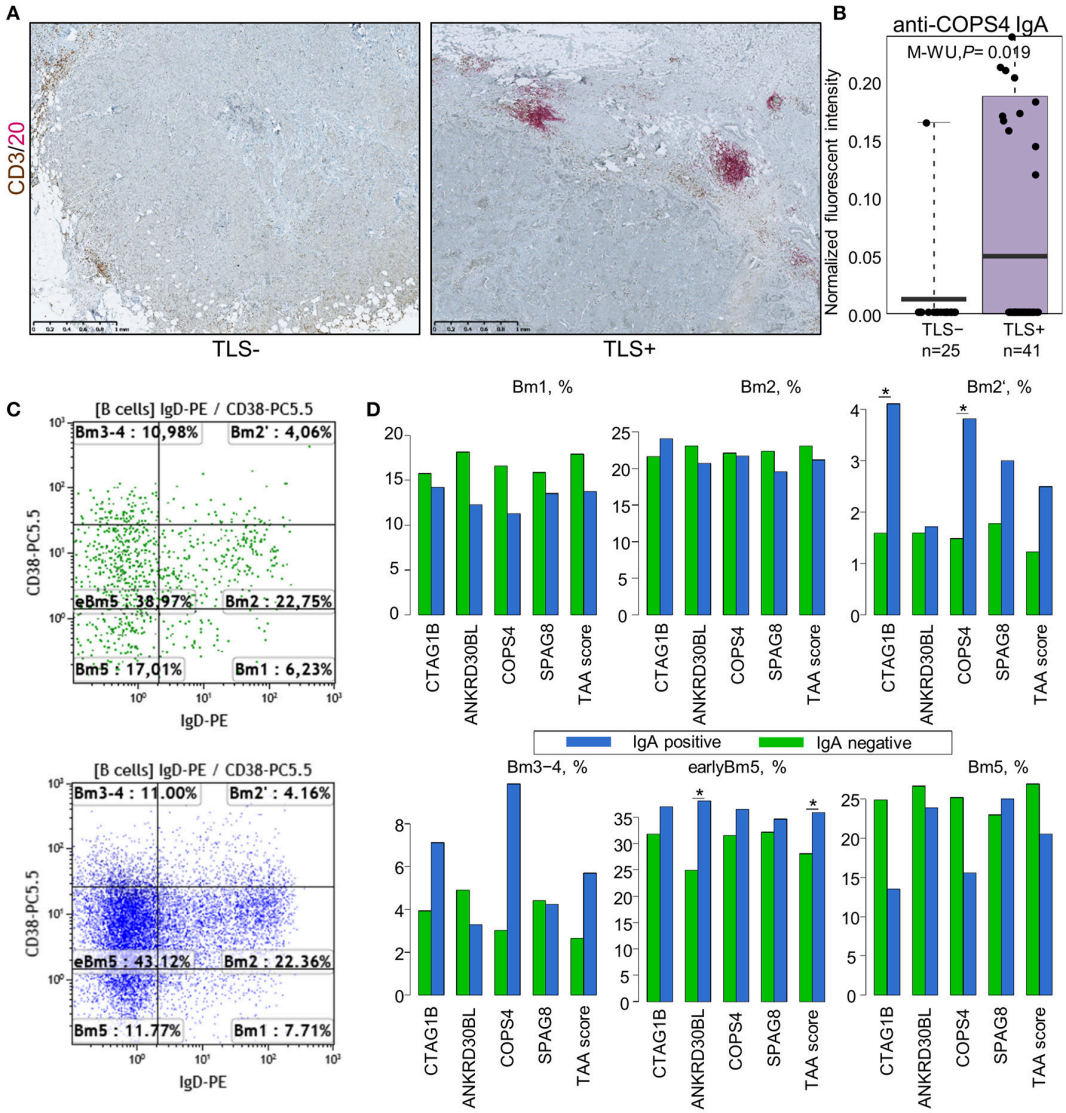
Association of IgA class autoantibodies with the immunological makeup of BC. **(A)** Representative images of TLS-negative (left) and TLS-positive (right) tumors determined by dual IHC using CD3 plus CD20. **(B)** Anti-COPS4 IgA levels in TLS-positive and TLS-negative tumors. **(C)** Representative FACS scatter-plots of B cell maturation stages in IgA-negative (upper) and IgA-positive (lower) tumors. **(D)** Percentage of B cells at various maturation stages in IgA-positive and IgA-negative SN-BC samples. Bm1, resting naïve; Bm2, activated naïve; Bm2′, pre-germinal center; Bm3-4, germinal center; eBm5, early memory; Bm5, late memory; *t-test, *P* < 0.05.

Unexpectedly, the levels of IgG or IgA antibodies against individual antigens or the TAA scores were not significantly different in groups of patients stratified by CD19^+^ B cell, PB/PC, CD4^+^ and CD8^+^ T cell density, and percentage of naïve and memory B cells (Figure S5). Similarly, no significant correlation was found by Spearman rank test. Mean PB/PC density tended to be higher in patients with anti-CTAG1B, AP1292 IgG and higher IgG-TAA score, and anti-CTAG1B, COPS4 IgA and higher IgA-TAA score in SN-BC, however the differences did not reach statistical significance (Figure S6). With the exception of anti-COPS4 IgA, the autoantibody levels were not significantly associated with the presence of TLS (Figure S5). The mean anti-COPS4 IgA level, however, was 7.5-fold higher (*P* = 0.019) in TLS-positive tumors (Figures 3A,B). Anti-COPS4 IgA was present in 11 out of 41 TLS+ tumors and only in 1 out of 25 TLS– tumors (OR 8.8, Fisher test *P* = 0.023). This suggests that the presence of TLS may be required for the production of some IgA specificities *in situ*. Furthermore, we found that the production of IgA but not IgG against 4 TAAs (CTAG1B, ANKRD30BL, COPS4, and SPAG8) as well as the IgA-TAA score was linked to the B cell maturation stages: the percentage of pre-germinal center (Bm2′ defined as CD19^+^IgD^+^CD38^hi^), germinal center (Bm3-4 defined as CD19^+^IgD^−^CD38^hi^) and early memory (eBm5 defined as CD19^+^IgD^−^CD38^+^) B cells was increased but the percentage of late memory B cells (Bm5 defined as CD19^+^IgD^−^CD38^−^) was similar or decreased in the IgA-positive as compared to IgA-negative BC samples (Figures 3C,D).

### The IgG-TAA score is associated with poor recurrence-free survival and lower tumor infiltrating CD8^+^ T cell counts

**Figure 4:**
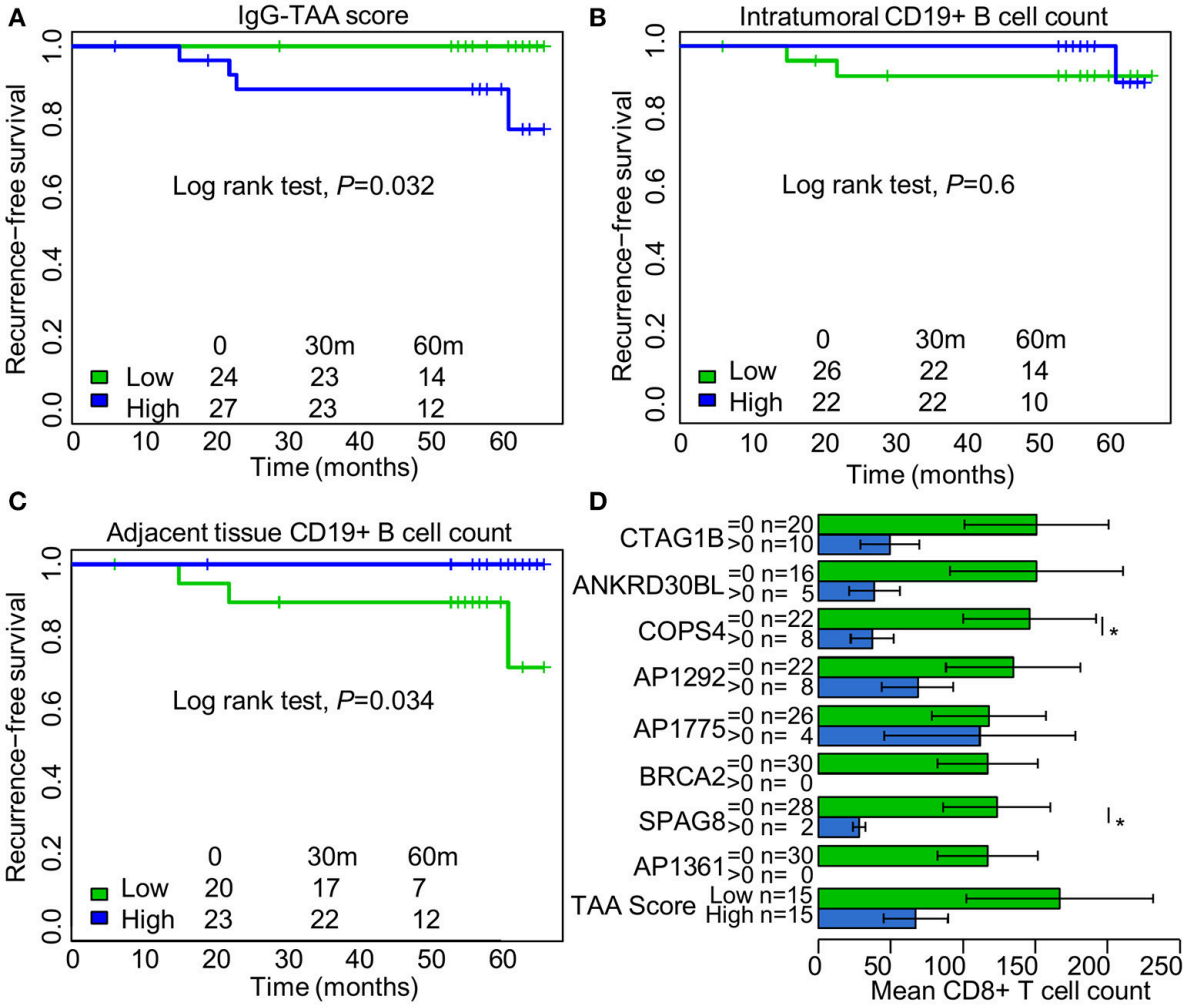
Prognostic significance of IgG class autoantibodies and association with tumor infiltrating CD8^+^ T cell counts. **(A)** Kaplan Meier plot showing the association between IgG-TAA score and RFS. **(B)** Kaplan Meier plot showing the association between intratumoral CD19^+^ B cell count and RFS. **(C)** Kaplan Meier plot showing the association between CD19^+^ B cell count in the adjacent tissues and RFS. **(D)** Tumor infiltrating CD8^+^ T cell counts in IgG-positive and IgG-negative SN-BC samples. *t-test, *P* < 0.05.

To assess the prognostic significance of the *ex vivo-*produced autoantibodies, the patients were separated into groups with high and low IgG and IgA-TAA scores using median as a threshold. Kaplan-Meier analysis was performed to assess the association of the scores with RFS. The analysis revealed that high IgG-TAA score is associated with shorter RFS (Log rank test, *P* = 0.032; Figure 4A), whereas the IgA-TAA score is not (Figure S7). At the same time, CD19^+^ B cell count within the tumor was not associated with RFS in our cohort (Figure 4B), while higher number of CD19^+^ B cells in the adjacent tissues was correlated with significantly increased RFS (Figure 4C).

To search for the possible reasons of the adverse prognostic significance of the IgG-TAA score, we compared the count of CD4^+^ and CD8^+^ T cells in groups of IgG-positive and negative samples. We found that the presence of anti-CTAG1B, ANKRD30BL, COPS4, SPAG8 antibodies, and high IgG-TAA score had a tendency to be associated with lower mean counts of CD8^+^ T cells, which reached statistical significance for anti-COPS4 and SPAG8 antibodies (Figure 4D). No such association was observed for IgA class antibodies.

## Discussion

To the best of our knowledge, this is the first study comprehensively examining the antigen specificity of antibodies produced *in situ* and correlating their presence with clinical and histological features and immunologic makeup of tumors. Furthermore, we simultaneously examined IgG and IgA responses thus providing the first insight into the prevalence and repertoire of IgA autoantibodies in BC patients and a head-to-head comparison of clinical significance of IgG and IgA responses.

Although some earlier studies suggested that non-lymphoid cells and cancers can produce immunoglobulin molecules (31, 32), a confirmation that these immunoglobulins are functional, affinity-matured antibodies capable of binding to tumor antigens is still lacking. Therefore, we assume that the autoantibodies analyzed in this study are produced by TIL-B.

We report here that over 84% of BC tissue specimens have autoantibodies against at least one antigen represented on our in-house generated 91-feature antigen array. The top three antigens most frequently recognized by the TIL-B autoantibodies are ANKRD30BL, COPS4, and CTAG1B. ANKRD30BL (Ankyrin repeat domain 30B like) is a poorly characterized protein that shows a partial similarity (65%, *E* value 1e-101) to ANKRD30A (also known as NY-BR-1)—a breast differentiation antigen that is frequently overexpressed in BC tissues (33) and has been shown to elicit spontaneous humoral and cellular immune response in BC patients (34). According to the NCBI's Gene database, ANKRD30BL expression in normal tissues is restricted to testis (35). Here, we for the first time show that it elicits BC-associated humoral immune response. Hence it may represent a novel CT antigen, however assessment of its expression pattern in tumor tissues is required in order to consider it as a potential immunotherapeutic target. COPS4 is a ubiquitously expressed intracellular protein, a subunit of COP9 signalosome that functions as an important regulator in multiple signaling pathways. We have previously found anti-COPS4 IgG in gastric and thyroid cancer and melanoma (19, 21), however the reasons for its immunogenicity are not clear. CTAG1B (also known as NY-ESO-1) is a well-known CT antigen eliciting humoral and cellular immune response in patients with various cancers including BC (25, 28, 36). Interestingly, ANKRD30BL predominantly elicited IgA response, while CTAG1B more frequently elicited IgG response. Autoantibodies against these antigens, however, were not strictly specific to BC and were also found in the normal breast tissues obtained from patients undergoing mammary reduction, though at lower frequencies. The only antigen showing strictly BC-specific response was AP1292—a 14 amino acid peptide encoded by a human intergenic DNA fragment sharing 77% identity with aminopeptidase of *Clostridium peptidivorans*. Of note, BC tissues have been recently shown to contain a variety of bacterial genera including *Clostridium* (37). Hence, AP1292 likely represents a mimotope of a bacterial antigen, which elicits B response specifically in BC patients. Moreover, the frequency of anti-AP1292 antibodies was significantly higher in tissues than in plasma. This suggests that *in situ* TIL-B responses may be directed not only against autoantigens but also against BC microbiome, however our antigen array technology does not allow a comprehensive characterization of such responses.

Comparison of the prevalence of autoantibodies in paired BC tissue and plasma samples showed that less than a half of patients had autoantibodies against the same antigen both in plasma and tissues. These results suggest that the *in situ*-produced autoantibodies and the respective TIL-B often remain sequestered in the tissue microenvironment and do not significantly contribute to the plasma autoantibody levels. This is in line with the observation that CD20^+^ TILs do not contribute significantly to the systemic level of anti-NY-ESO-1 and TP53 antibodies in patients with ovarian cancer (38). The opposite was also true, the presence of given autoantibodies in the circulation was not indicative of the autoantibody production *in situ*. Whether such B cells were activated in draining lymph nodes as it has been recently demonstrated for anti-CTAG1B B cells in BC patients (39) and never infiltrated the tumors or whether they were activated in BC-associated TLS but left the tissue is not known.

We and others have reported that BC tissues contain substantial amounts of IgA immunoglobulins (15, 23), however the antigen specificity and biological functions of these antibodies remained unknown. Here we show that IgA present in the BC tissues recognize BC-associated antigens as well as cancer-non-related antigens and the overall prevalence of IgA is similar to that of IgG class autoantibodies. However, the IgA and IgG responses were not concordant and their presence was associated with distinct clinical and histological features.

We found that high IgG-TAA score (but not IgA-TAA score) is associated with shorter RFS in our cohort of BC patients. This finding is in line with a number of other studies showing that systemic IgG responses to tumor-associated antigens are associated with poor prognosis and shorter overall or progression free survival in patients with melanoma (40), gastric cancer (41), hepatocellular carcinoma (42) and breast cancer (43). Our data suggest that the presence of *in situ* IgG responses is associated with more aggressive pathologic features, such as HER2 overexpression and later stage of BC, as well as lower mean counts of tumor infiltrating CD8^+^ T cells, however the causal relationship between the IgG responses and these features remains unclear. One possible explanation is that the cytokine/chemokine profile in the tumor microenvironment that promotes IgG production inhibits the cytotoxic function of T cells, hence the presence of IgG may be a marker of Th2-polarized microenvironment. Another possibility is that IgG have tumor-promoting properties by themselves. For example, IgG have been shown to promote cancer development by activating Fc-γ receptors on myeloid cells that in turn regulate recruitment, composition, and effector functions of TILs in a mouse model of squamous carcinogenesis (44). Furthermore, IgG4 subclass autoantibodies have been found to impair IgG1-mediated antitumor immunity and to promote Th2-biased inflammation in melanoma (45). Alternatively, the prognostic significance may be related to the expression of the respective antigens not the presence of autoantibodies itself. It also has to be noted that the presence and intensity of the IgG analyzed in this study was not directly proportional to the density of TIL-B. Moreover, the count of intratumoral TIL-B was not associated with RFS in our cohort, whereas higher number of peritumoral TIL-B was associated with better RFS.

It's also possible that only a minor fraction of CD19^+^ TIL-B secretes antibodies and the antibody production may not be their main effector function. Alternatively, TIL-B may serve as antigen-presenting cells, secrete polarizing cytokines and foster the formation of TLS as it has been shown in serous ovarian cancer and lung squamous cell carcinoma (46–48) and more recently in BC (submitted for publication by Garaud et al. submitted).

We hypothesized that the production of autoantibodies *in situ* may be associated with the formation and maturation of TLS in the tumor microenvironment, therefore we correlated the presence of IgG and IgA with the presence of TLS and phenotypic features of TIL-B. Since we have previously shown that the development of tumor-associated TLS follows sequential stages analogous to the formation of germinal centers (GC) in lymph nodes (48), we classified the TIL-B as resting naïve, activated naïve, pre-GC, GC, early memory, and late memory B cells according to the B cell maturation stages during the formation of GCs in lymph nodes. Our data show that the proportion of pre-GC, GC, and early memory B cells is increased in tumors with IgAs against BC-associated antigens and high IgA-TAA scores as compared with IgA-negative tumors. Furthermore, the level of anti-COPS4 IgA was significantly higher in tumors with TLSs as compared with TLS-negative tumors. Whereas, no association of IgG-class antibodies with the presence of TLS or B cell maturation stages was found. These results suggest that the activation of naïve B cells and Ig class switching to IgA may take place in the TLS. These findings are in an agreement with a study by Cipponi et al. showing that TLS-associated B cells found in the metastatic lesions of melanoma undergo clonal amplification and somatic hypermutations, and produce IgM, IgG, and IgA class antibodies (49). However, why only anti-COPS4 IgA but no other antibodies correlated with the presence of TLS in our cohort is unclear. One explanation of this observation could be that the formation of TLS is highly dynamic and the structures are short-lived, thus obscuring the link between the presence of TLS and autoantibodies. Alternatively, it could be possible that only a fraction of TIL-B is *de novo* activated in the TLS, while other antigen-experienced B cells have migrated into the tumor tissue from secondary lymphoid organs, and thus produce antibodies independently of TLS. Finally, the absence of correlation with the presence of TLS could be explained by the concept of T cell-independent antigens. The production of antibodies in the absence of T cell help is largely mediated by marginal zone B cells, which differentiate into short-lived plasma cells, with limited isotype switching to some IgG subtypes and also to IgA (50, 51).

In summary, this study for the first time examined the antigen specificity of antibodies produced by TIL-B and showed that they are directed against BC-associated antigens, BC non-specific autoantigens and possibly also BC-associated microbial antigens. Their frequency in BC tissues is higher than that in normal breast tissues and their production *in situ* is uncoupled from systemic humoral responses. Although the prevalence of IgG and IgA class autoantibodies was similar, IgG and IgA responses were not concordant and they correlated with distinct clinical, pathological and immunological features possibly reflecting distinct signals required for their production and different biological functions in the tumor microenvironment.

## Author contributions

KW-G, KS, SG, LB, and AL designed research, SG, KS, PZ, and UR performed research and participated in analysis and interpretation of the results, PZ performed the microarray data analysis and statistical analyses, SG and LB collected clinical data, GV and DL provided human samples, AdW and GV performed the pathological evaluations, AL wrote the manuscript, KW-G, KS, and SG revised the manuscript. All authors have read and approved the manuscript.

### Conflict of interest statement

The authors declare that the research was conducted in the absence of any commercial or financial relationships that could be construed as a potential conflict of interest.
