# Supplementary figures and images for: Antigen Specificity and Clinical Significance of IgG and IgA Autoantibodies Produced in situ by Tumor-Infiltrating B Cells in Breast Cancer
Source: Front Immunol. 2018 Nov 20;9:2660. doi: 10.3389/fimmu.2018.02660 (PMC6255822; doi:10.3389/fimmu.2018.02660)

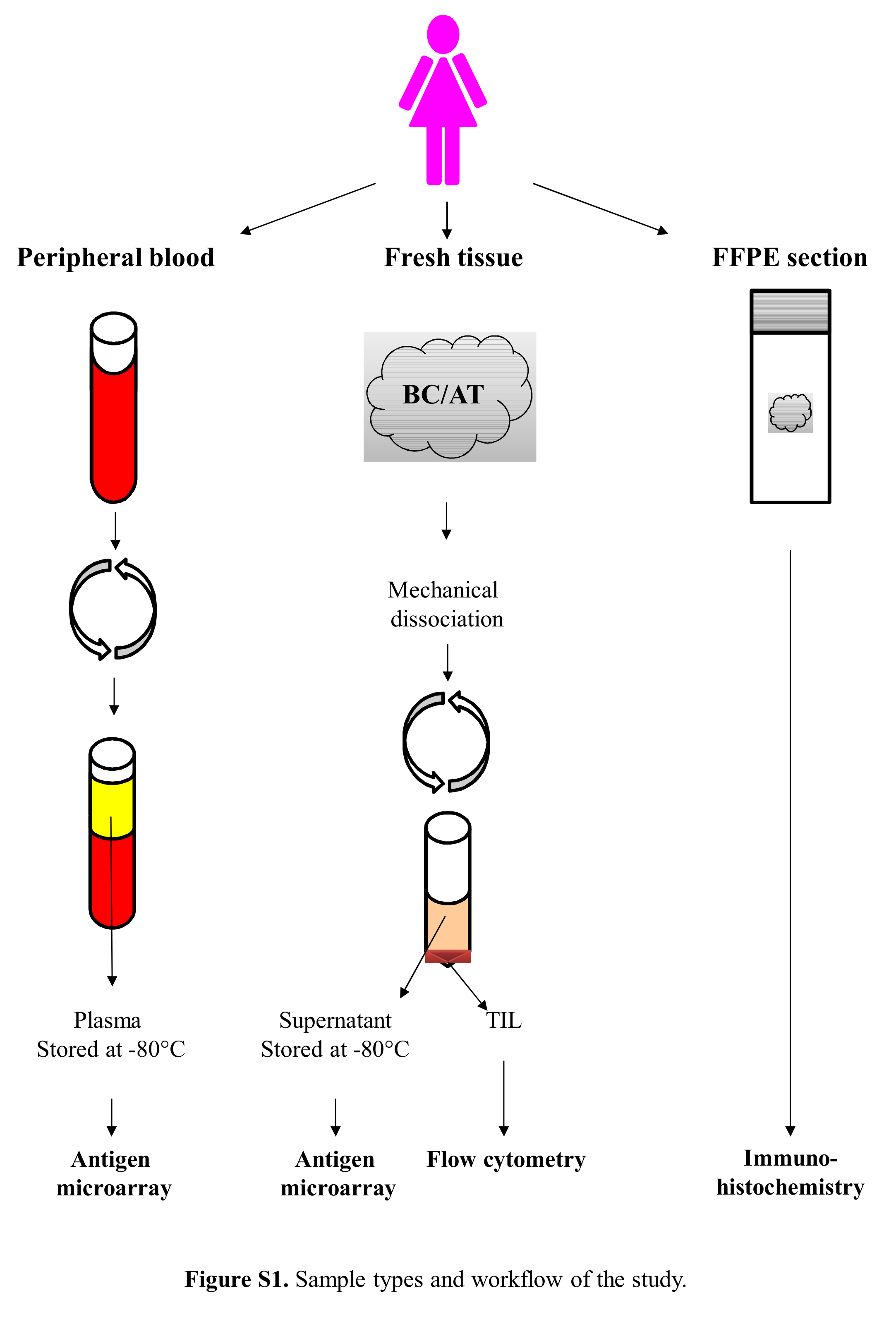

Supplement: Supplementary file 3 [file Image_1.TIF]

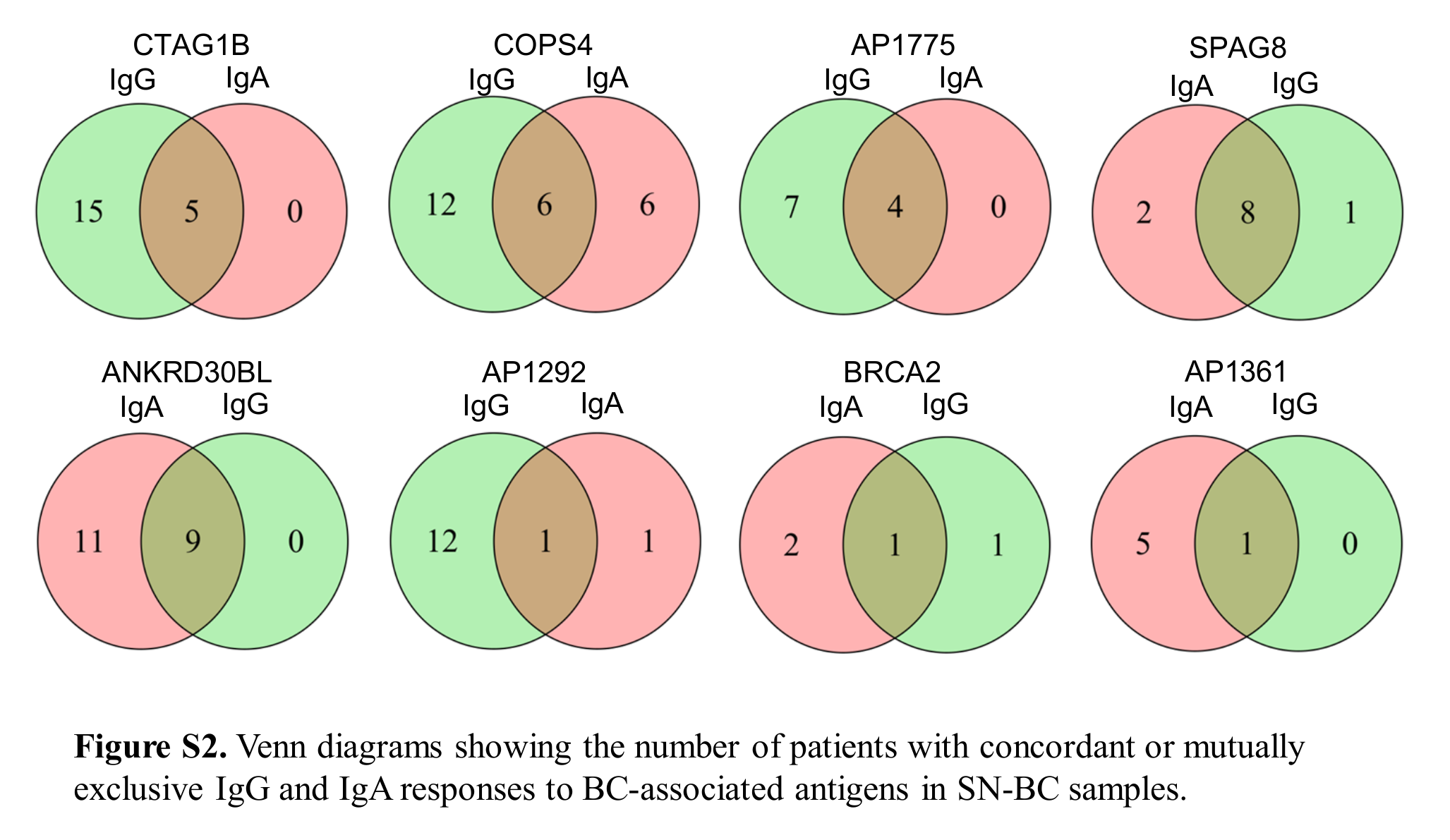

Supplement: Supplementary file 4 [file Image_2.TIF]

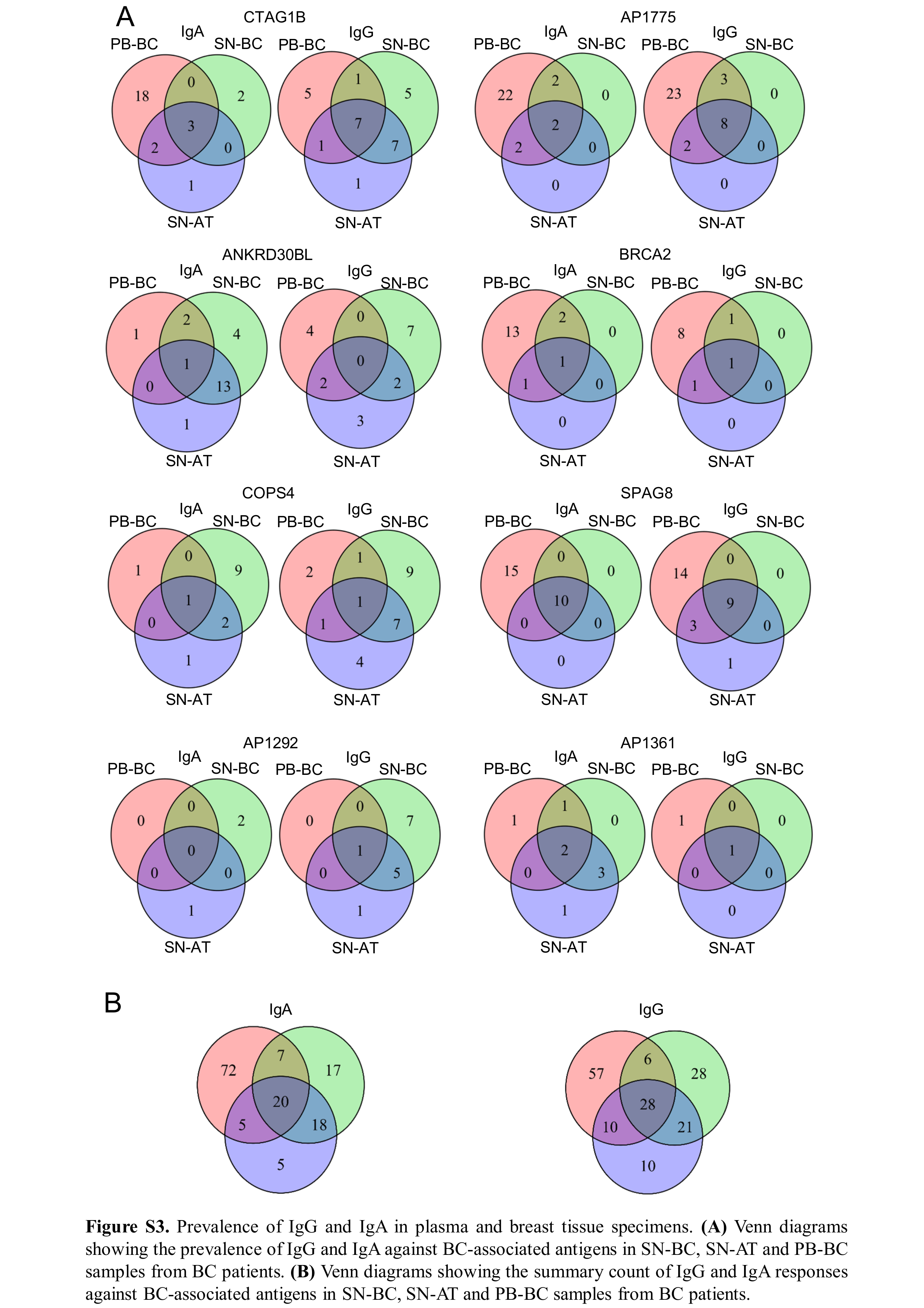

Supplement: Supplementary file 5 [file Image_3.TIF]

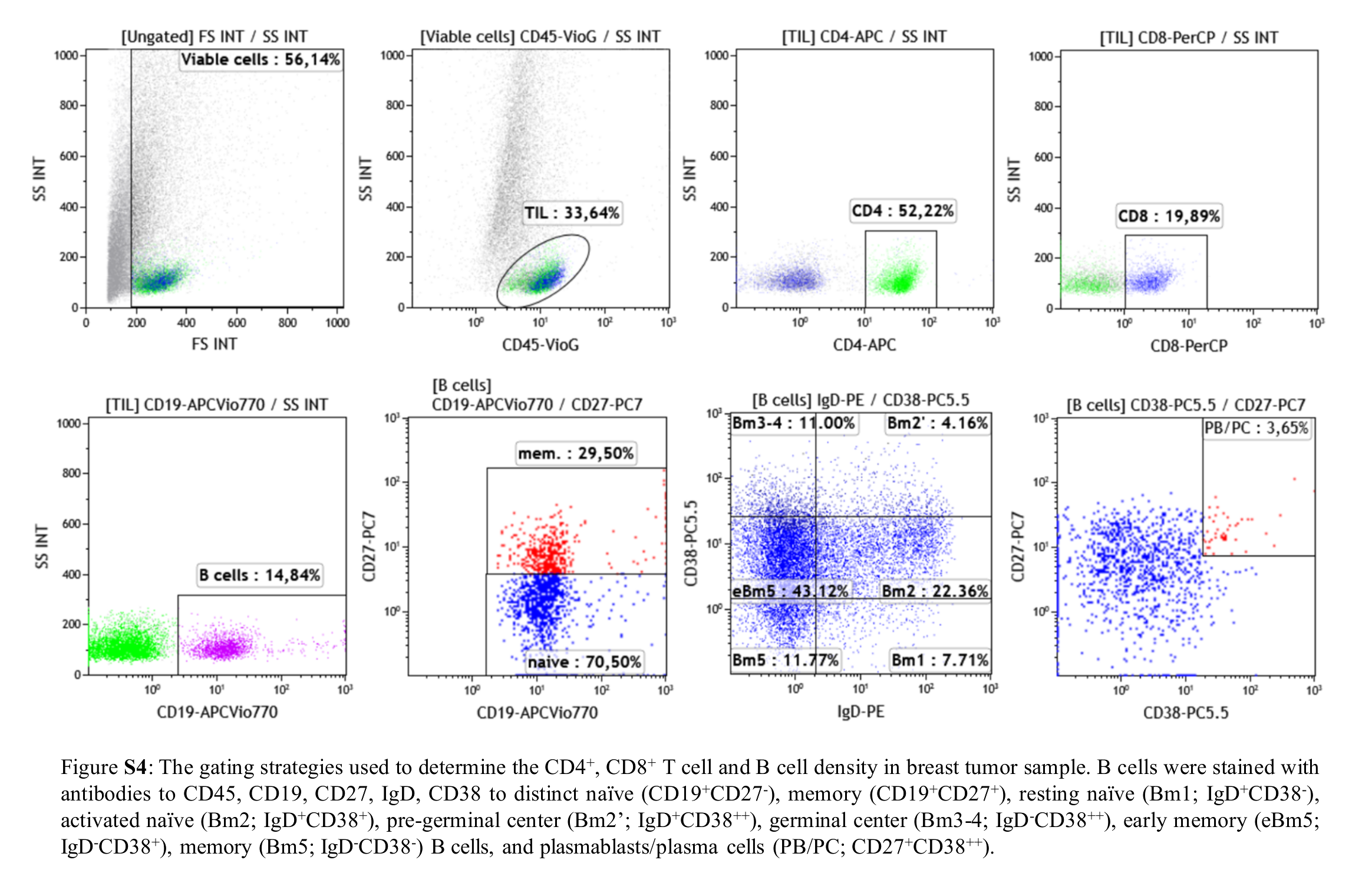

Supplement: Supplementary file 6 [file Image_4.TIF]

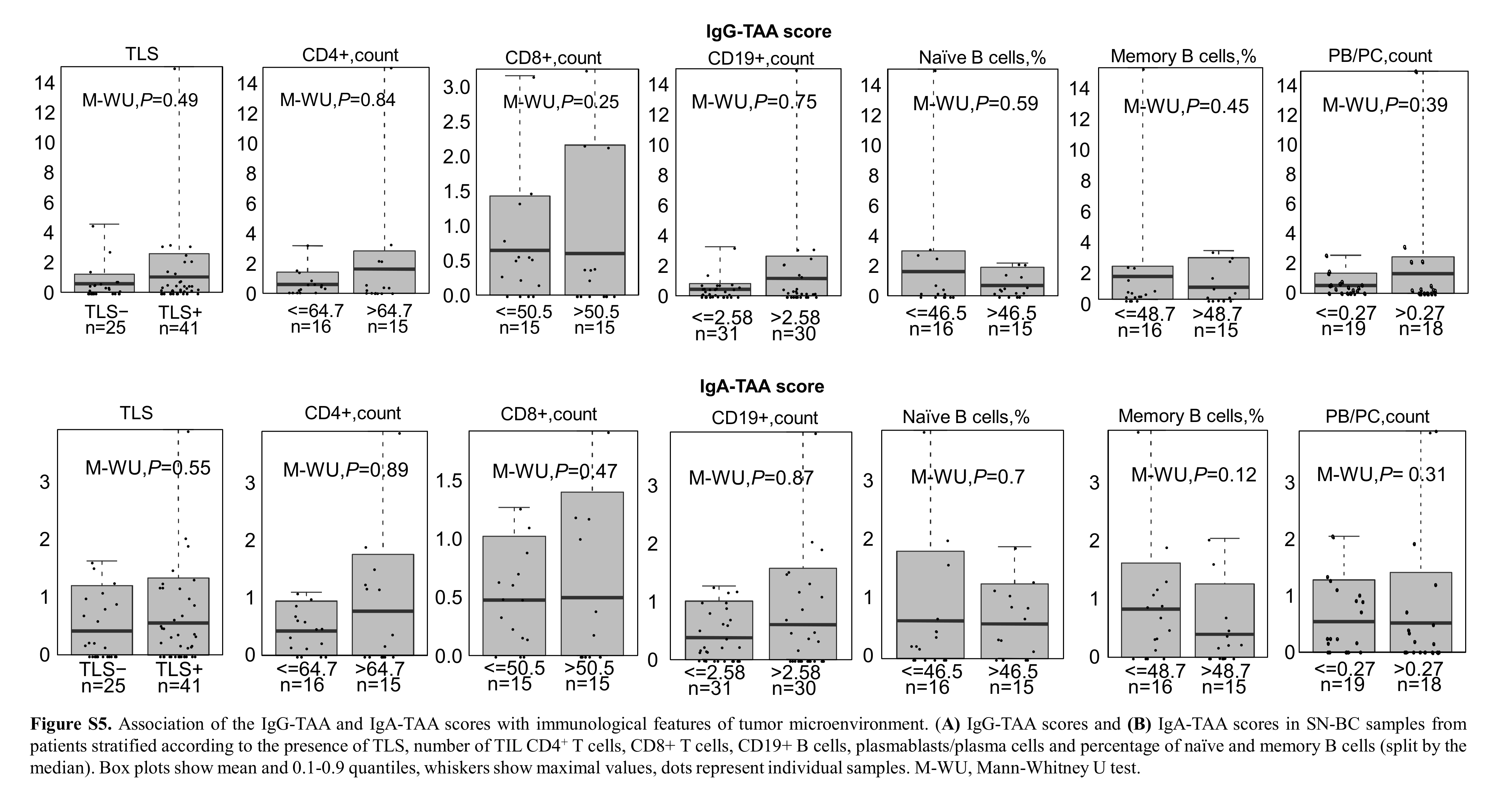

Supplement: Supplementary file 7 [file Image_5.TIF]

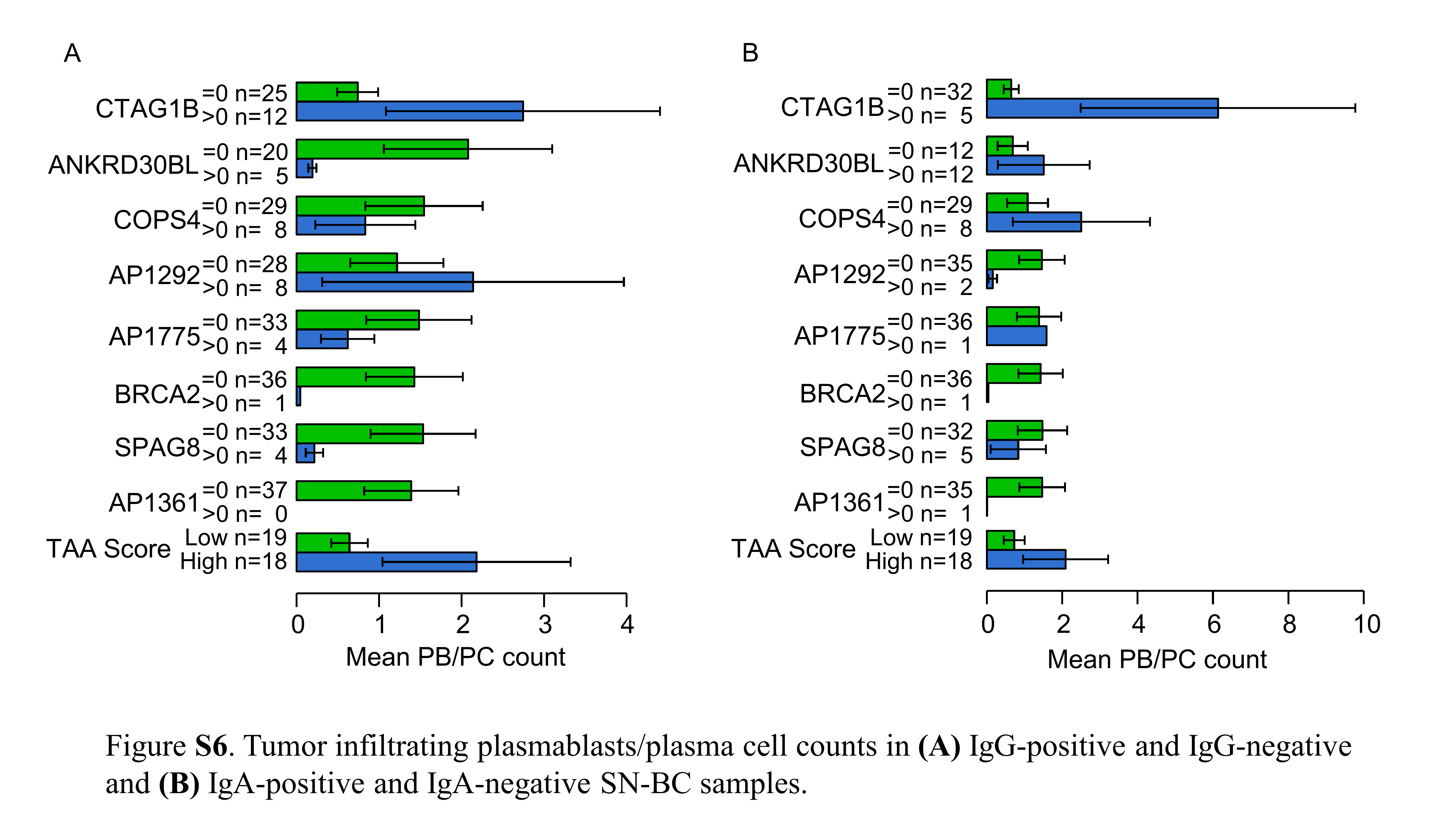

Supplement: Supplementary file 8 [file Image_6.TIF]

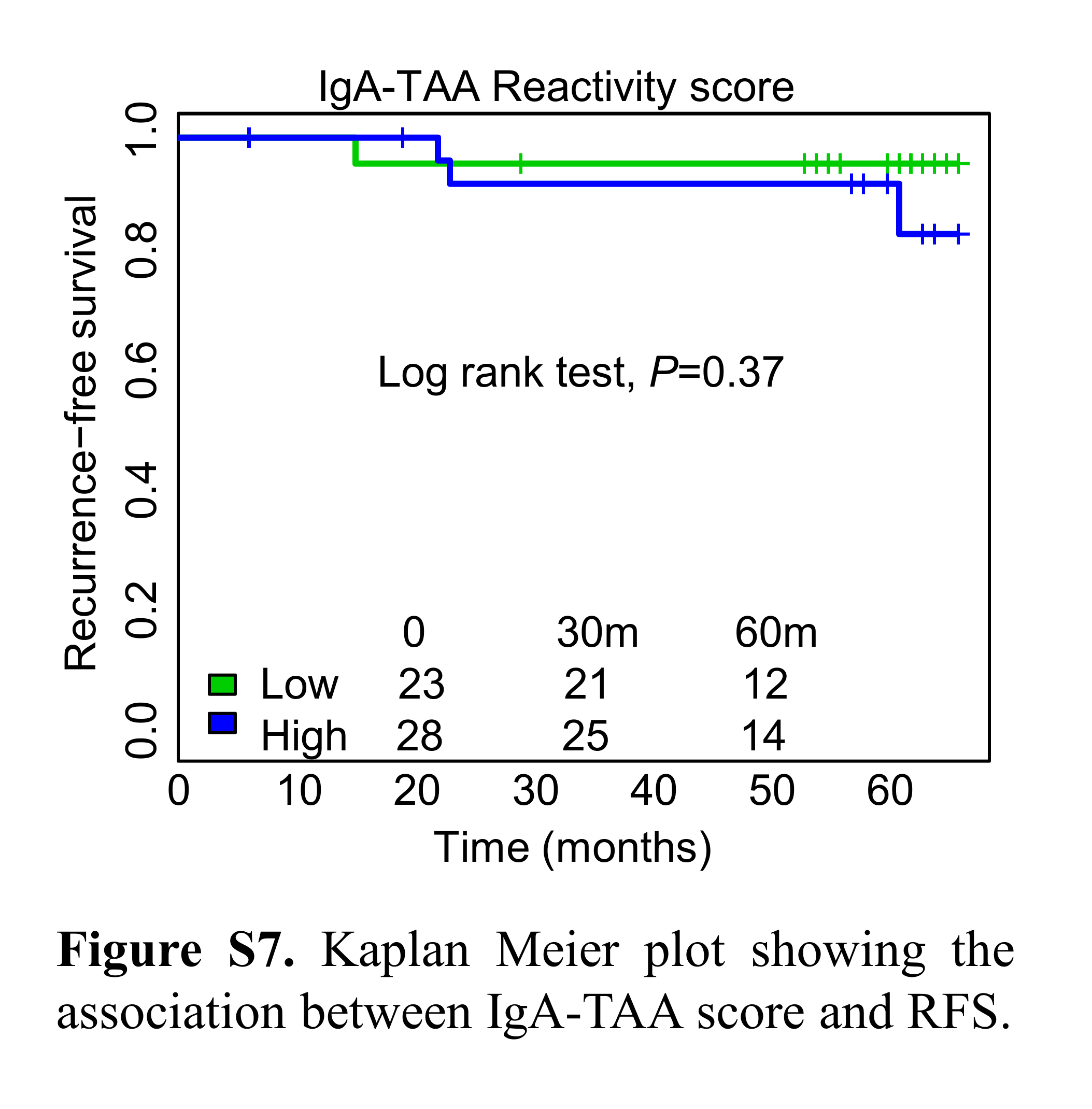

Supplement: Supplementary file 9 [file Image_7.TIF]
